# Identification of differentially expressed proteins in the injured lung from zinc chloride smoke inhalation based on proteomics analysis

**DOI:** 10.1186/s12931-019-0995-0

**Published:** 2019-02-15

**Authors:** Xiaowei Xie, Jingan Zhao, Lixin Xie, Haiyan Wang, Yan Xiao, Yingjia She, Lingyun Ma

**Affiliations:** 1 Medical School of Chinese PLA, Medical School of Chinese PLA, Fuxing Road, Beijing, 100853 China; 2 Department of Respiratory, The Fourth Medical Center of Chinese PLA General Hospital, Beijing, China; 3 Department of Pulmonary and Critical Care Medicine, Chinese PLA General Hospital, Beijing, China

**Keywords:** Smoke inhalation, Lung injury, iTRAQ, WGCNA, Immunohistochemistry, Differentially expressed proteins, Signalling pathways

## Abstract

**Background:**

Lung injury due to zinc chloride smoke inhalation is very common in military personnel and leads to a high incidence of pulmonary complications and mortality. The aim of this study was to uncover the underlying mechanisms of lung injury due to zinc chloride smoke inhalation using a rat model.

Methods: Histopathology analysis of rat lungs after zinc chloride smoke inhalation was performed by using haematoxylin and eosin (H&E) and Mallory staining. A lung injury rat model of zinc chloride smoke inhalation (smoke inhalation for 1, 2, 7 and 14 days) was developed. First, isobaric tags for relative and absolute quantization (iTRAQ) and weighted gene co-expression network analysis (WGCNA) were used to identify important differentially expressed proteins. Gene Ontology (GO) and Kyoto Encyclopedia of Genes and Genomes (KEGG) pathway analyses were used to study the biological functions of differentially expressed proteins. Then, analysis of lung injury repair-related differentially expressed proteins in the early (day 1 and day 2) and middle-late stages (day 7 and day 14) of lung injury after smoke inhalation was performed, followed by the protein-protein interaction (PPI) analysis of these differentially expressed proteins. Finally, the injury repair-related proteins PARK7 and FABP5 were validated by immunohistochemistry and western blot analysis.

**Results:**

Morphological changes were observed in the lung tissues after zinc chloride smoke inhalation. A total of 27 common differentially expressed proteins were obtained on days 1, 2, 7 and 14 after smoke inhalation. WGCNA showed that the turquoise module (which involved 909 proteins) was most associated with smoke inhalation time. Myl3, Ckm, Adrm1 and Igfbp7 were identified in the early stages of lung injury repair. Gapdh, Acly, Tnni2, Acta1, Actn3, Pygm, Eno3 and Tpi1 (hub proteins in the PPI network) were identified in the middle-late stages of lung injury repair. Eno3 and Tpi1 were both involved in the glycolysis/gluconeogenesis signalling pathway. The expression of PARK7 and FABP5 was validated and was consistent with the proteomics analysis.

**Conclusion:**

The identified hub proteins and their related signalling pathways may play crucial roles in lung injury repair due to zinc chloride smoke inhalation.

## Introduction

Military operations are complex and are always very dangerous because of toxic industrial chemicals and materials such as smoke bombs. Smoke bombs generate large amounts of particles and toxic gases during combustion and explosion. Therefore, lung smoke inhalation injuries are common in military personnel. It has been noted that smoke inhalation is the leading cause of lung injury [1]. In November 2014, 15 soldiers sustained lung injuries during smoke bomb inhalation [2, 3]. In addition, 3 soldiers sustained lung injuries during smoke bomb inhalation, and one of the soldiers died from the severe lung injury in January 2017 [4].

Generally, lung injury from smoke inhalation is defined as the inhalation of thermal or chemical irritants (such as zinc chloride), with a very high incidence of pulmonary complications and mortality [5–7]. A cough, difficulty breathing, nausea, vomiting and abnormal liver function are the main symptoms of lung injuries due to smoke inhalation. Despite many years of intense research, the molecular mechanisms involved in the pathology of lung injury from smoke inhalation are poorly illuminated [1, 2, 5]. Rats are model animals suitable for studying the effects of inhaled smoke on lung injury [8]. To study the potential mechanisms of smoke inhalation on lung injury, we developed a rat model with high doses of zinc chloride smoke inhalation to cause lung injury.

It is noted that isobaric tags for relative and absolute quantization (iTRAQ), mass spectrometry-based quantitative approaches and weighted gene co-expression network analysis (WGCNA) have largely contributed to proteomics investigations [9]. Applying the above techniques, we first analysed the differentially expressed proteins from days 1, 2, 7 and 14 after zinc chloride smoke inhalation in our rat model. Then, we further identified lung injury repair-related, differentially expressed proteins in the early and middle-late stages of lung injury. Our study may be helpful in understanding the potential molecular mechanisms in lung injuries due to smoke inhalation.

## Materials and methods

### The lung injury rat model due to smoke inhalation

Male Wistar rats with a weight of 200 g were supplied by the Academy of Military Medical Sciences of the PLA and were raised carefully in accordance with the ethical guidelines of the National Institutes of Health on animal care. All experimental procedures were approved by the Academy of Military Medical Sciences of the PLA. The homemade smoke chamber consisted of 5 g of smoke bomb materials (aluminium powder, zinc oxide, hexachloroethane, silicon powder and black powder). The smoke bomb was placed into a special rectangular iron pot with a diameter of 2 cm, a magnesium strip was placed in the pot to ignite, and the smoke bomb was burned for approximately 45 s. The smoke chamber is 1 m long, 0.8 m wide, 0.6 m high and 0.48 m^3^ in volume. The smoke box allows samples to be taken quickly, and these samples are measured by a gas sampler to determine the concentration of the individual smoke components in the gas. The concentration of the 5-g smoke bombs was 10.625 g.m-3. The gas sampling method was used to determine the concentration of the zinc ions in the smoke generated by the smoke bomb, and a concentration of 4.996 mg/L was determined. The male Wistar rats were randomly divided into the control group (*n* = 8) or the exposed group (*n* = 164), and the male Wistar rats in the exposed group were only exposed once on the first day for 5 min, and then the smoke was cleared for 10 min. In this study, the animals were only exposed once to a high dose because all human exposures are high-dose accidents and are not repeated occupational exposures. Lung tissues were collected 1, 2, 7, 14, 21 and 28 days after the initial exposure.

### Histopathology

The right, upper lobe of the lung was fixed in 10% formalin, dehydrated, embedded in paraffin and cut into 6 μm sections. After deparaffinization, the tissues were haematoxylin and eosin (H&E) stained or Mallory stained, and the morphological lesions and changes in lung tissues were observed under a light microscope.

### Proteomics analysis

Lung tissues were harvested and lysed in RIPA lysis buffer with protease inhibitors for 30 min at 4 °C. Lysates were then centrifuged for 10 min at 12000 rpm at 4 °C, and the insoluble debris was discarded. The protein in the lung tissues was extracted, followed by alkylation treatment. The concentration test of the extracted protein was then performed. Sodium dodecyl sulfate was used for protein electrophoresis [10, 11]. Protein digestion was conducted using the filter-aided sample preparation (FASP) method described in Wisniewsk et al. [12]. After protein enzymolysis, the iTRAQ reagent was applied to mark peptides [10]. iTRAQ labelling was performed by using the iTRAQ reagent-8plex Multiplex Kit (AB Sciex California, USA) according to the manufacturer’s instructions. Strong cation exchange chromatography was used to predissociate a balanced mixture of marked peptides [13]. The pooled samples were analysed by 2D LC-MS/MS. LC–electrospray ionization (ESI)–MS/MS analysis was conducted on a liquid chromatography machine coupled with tandem mass spectrometry (nLC, Thermo Fisher Scientific). A 10-μL aliquot of each fraction was injected for 2D LC-MS/MS analysis. A mass spectrometer (MS), (Orbitrap Fusion, Thermo Scientific) was used to analyse the eluted peptides. The MS data were acquired with the Orbitrap detector by using the fastest mode. For database searching, the differentially expressed proteins were identified with an abundance ratio ≥ 1.5 or an abundance ratio < 0.667 and *p*-values≤0.05. A heat-map analysis of the differentially expressed protein expression was performed by using pheatmap in R.

### Construction of the WGCNA co-expression network

To find significant modules and proteins in the proteome data, the WGCNA package in the R software package (http://www.r-project.org/) was used to construct the co-expression network of the proteins. The rationale behind the WGCNA approach is that highly correlated genes, in terms of their expression values, are very likely to work in the same biological processes and/or pathways so that they can be grouped into a common network module [14]. The type of network is signed, which allows positively or negatively regulated genes to be grouped into different network modules. First, Pearson’s correlation matrices were calculated between pairwise genes [15]. Then, the similarity matrix was transformed into an adjacency matrix (AM) using a power β = 10 based on the scale-free topology criterion described in the WGCNA package documents [16]. Afterwards, the adjacency matrix was transformed into a topological overlap matrix and was, in turn, converted into a dissimilarity topological overlap matrix, from which a dendrogram was mapped via hierarchical clustering. The clusters were obtained from the dendrogram by using the dynamic tree cutting technique [17]. The next step was to further identify the most important modules for analysis. We first calculated the eigengene of each module, which is defined as the first principal component of its representation matrix. Then, we used these eigengenes to compute module-smoke inhalation time associations. The module-smoke inhalation time association for a given module and smoke inhalation time is the correlation between the eigengene of the module and the smoke inhalation time. Lastly, we chose six modules in association with the smoke inhalation times. In addition, to explore the association between the modules and the smoke inhalation times, a correlation analysis was performed. The modules with absolute values of correlation coefficients > 0.8 and *p* < 0.001 were identified to be smoke inhalation time-associated modules. The Cytoscape software (http://cytoscape.org/) was used for the visualization of the most significant time-associated module with a threshold > 0.5. Cytoscape cores are available for layout, scripting, file formatting and linking the network to databases [18]. Nodes represent proteins, and edges represent interactions between two proteins [19]. The pivotal nodes in the network were identified based on their degrees of connectivity.

### Functional analysis of proteins in the turquoise module

To study the biological function of proteins in the turquoise module, Gene Ontology (GO) and Kyoto Encyclopedia of Genes and Genomes (KEGG) pathway analyses were performed by using the online software GeneCodis3 (http://genecodis.cnb.csic.es/analysis). The false discovery rate (FDR) value of each enriched pathway was assigned with -log10 transformation. The threshold of the false discovery rate (FDR) < 0.05 was set as the criteria for statistical significance.

### Identification of hub proteins in the turquoise module

To further obtain key proteins, we screened hub differentially expressed proteins in the turquoise module. The proteins with Coefficient.Time + Coefficient.turquoise> 0.9, p.Time + p.turquoise< 0.01 were identified to be hub proteins. A heat-map analysis of the expression of hub proteins was performed by using pheatmap in R language.

### Lung injury repair-related protein analysis in the early and middle-late stages of zinc chloride smoke inhalation

To identify potential injury repair-related proteins and related signalling pathways in the early (day 1 and day 2) and middle-late (day 7 and day 14) stages of lung injury after smoke inhalation, we analysed the differentially expressed proteins on day 1 and day 2 compared with the control and on day 7 and day 14 compared with the control, respectively. All these key differentially expressed proteins intersected with those in the most significant smoke time-associated module.

### Protein-protein interaction analysis

To understand the protein interaction between key differentially expressed proteins in the early (4 differentially expressed proteins) and middle-late (96 differentially expressed proteins) stages of smoke inhalation, the STRING database (http://www.string-db.org/) was utilized to select interacting protein pairs. Through the PPI relationship obtained in the previous step, the protein-protein network (PPI) was visualized by Cytoscape software (http://cytoscape.org/). The degree was analysed by Cytoscape software. In the network, the nodes represent proteins, and the edges represent the interactions between the two proteins.

### Immunohistochemistry validation of differentially expressed injury repair-related proteins

In this study, PARK7, a differentially expressed protein on day 15 after smoke inhalation, was selected for immunohistochemistry (IHC) analysis on days 1, 2, 7, 14, 21 and 28. Then, 5 μm thick continuous sections were incubated with an anti-PARK7 antibody produced in rabbit (1:200 dilution; Abcam) followed by goat anti-rabbit immunoglobulin antibody conjugated by horseradish peroxidase (1:200 dilution; Vector). Then, the slides were visualized using diaminobenzidine (DAB) substrate (Vector). The number of positive cells was calculated under the microscope.

### Western blot analysis of differentially expressed injury repair-related proteins

In this study, the differentially expression proteins PARK7 and FABP5 after day 14 of smoke inhalation were selected for western blot (WB) analysis on days 1, 2, 7 and 14. Protein extracts were prepared by homogenization with protein lysates. Protein concentrations were quantified with an Enhanced BCA Protein Assay Kit (Thermo Fisher Scientific, Rochester, N.Y., USA). Protein samples were run on a 12% sodium dodecyl sulfate polyacrylamide gel for electrophoresis and were transferred to a polyvinylidene fluoride membrane. The membrane was blocked with non-fat dried milk for 1 h and was incubated with the following primary antibodies: rabbit anti-human FABP5 (1:1000 dilution), rabbit anti-human PARK7 (1:500 dilution) and mouse monoclonal IgG1 to GAPDH (1:3000 dilution). Primary antibody binding was detected using horseradish peroxidase-conjugated secondary antibody (1:10000 dilution). Detection was achieved with the ECL chemiluminescence kit (Pierce). Bands from three separate western blots were analysed by Quantiscan software (Biosoft, Great Shelford, Cambridge, UK).

### Statistical analysis

Immunohistochemistry and western blot statistical analyses were performed using GraphPad Prism (GraphPad Software, La Jolla, CA). Student’s t-test was used to assess statistical significance. Statistical significance was ascribed to a *p* value < 0.05. Graphs are presented as the mean ± SD. Immunohistochemistry and western blot experiments were repeated independently at least three times.

## Results

### Histopathology analysis of rat lungs after zinc chloride smoke inhalation

**Fig. 1:**
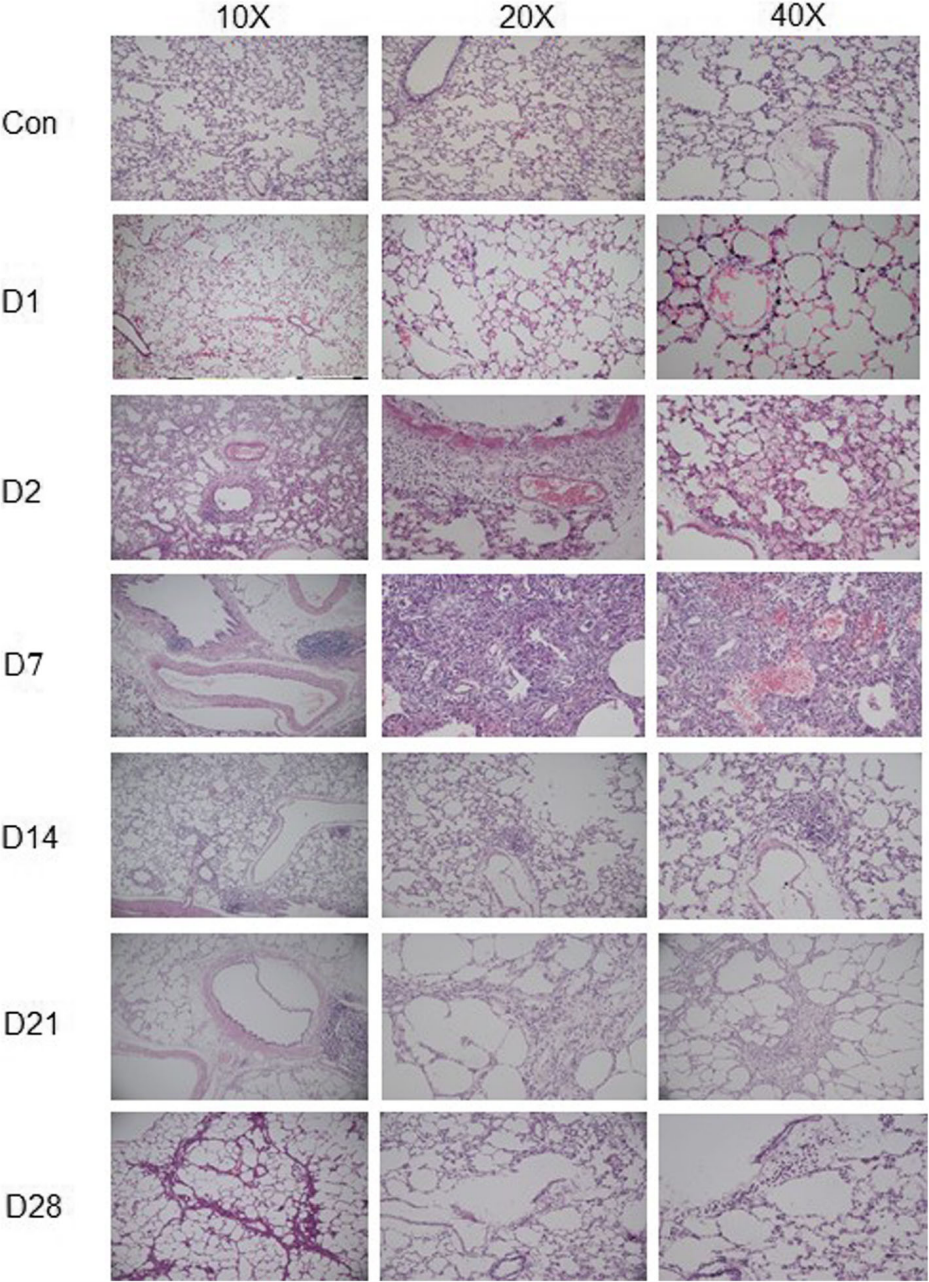
Histopathology analysis of rat lung on day 0, 1, 7, 14, 21 and 28 of smoke inhalation

The histopathology analysis results of rat lungs after zinc chloride smoke inhalation are shown in Fig. 1. The control group showed a normal lung structure. On day 1 after smoke inhalation, vascular wall thickening, oedema, a large number of black particles around the lumen, and neutrophilic granulocytes increased. On day 2 after smoke inhalation, exudative pneumonia, a large amount of exudation in the alveolar cavity and infiltration of lymphocytes around the bronchioles were observed. On day 8 after smoke inhalation, bleeding, emphysema, obvious cavities around the blood vessels, fibroblast proliferation, and massive lymphocytes in the alveolar cavity were observed. On day 14 after smoke inhalation, the alveolar cavity was obviously dilated, and scattered inflammatory foci were visible. On day 21 after smoke inhalation, the bronchial wall dilated and developed into an interstitial emphysema. The alveolar sac was extremely dilated, the interstitial sac was obviously widened, and there was a large amount of inflammatory cell infiltration. On day 28 after smoke inhalation, fibrous tissue hyperplasia, pulmonary fibrosis, eosinophilic infiltration of alveolar cavities, and alveolar expansion were observed.

### Mallory staining analysis of rat lungs after smoke inhalation

**Fig. 2:**
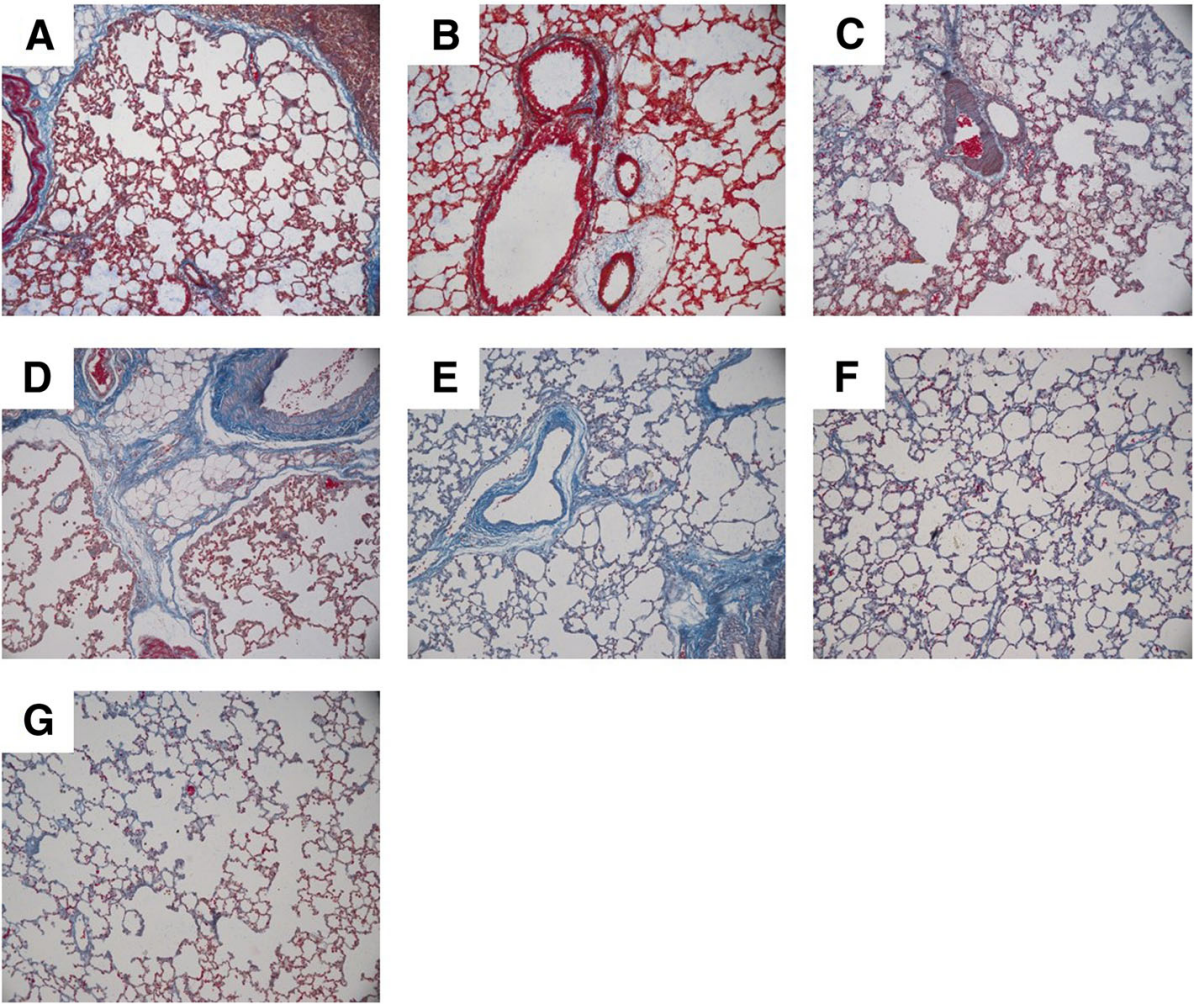
Mallary staining analysis of rat lung on day 0, 1, 7, 14, 21 and 28 of smoke inhalation. **a** Control. **b** Day 0 of smoke inhalation. **c** Day 1 of smoke inhalation. **d** Day 7 of smoke inhalation. **e** Day 14 of smoke inhalation. **f** Day 21 of smoke inhalation. **g** Day 28 of smoke inhalation

The Mallory method was used for tricolour staining of lung tissues. The Mallory staining results of rat lungs after smoke inhalation are shown in Fig. 2. The dark blue substance was significantly increased in lung tissues on days 1 (B), 2 (C), 7 (D), 14 (E), 21 (F) and 28 (G) after smoke inhalation compared with that in the control group (A), indicating that pulmonary fibrosis occurred after smoke inhalation.

### Identification of differentially expressed proteins

**Fig. 3:**
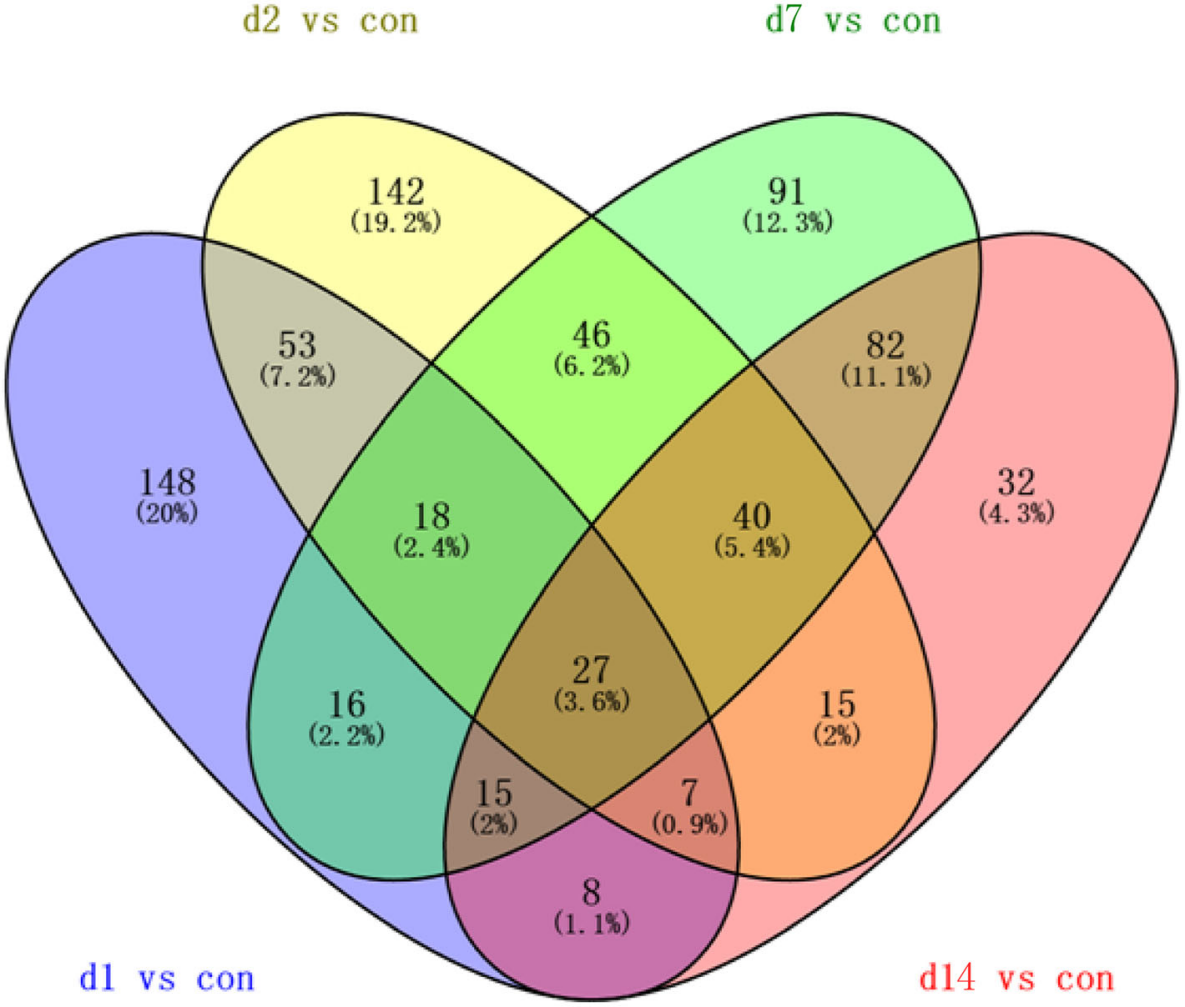
The Venn diagram of differentially expressed proteins on day 1 vs control, day 2 vs control, day 7 vs control, and day 14 vs control

**Fig. 4:**
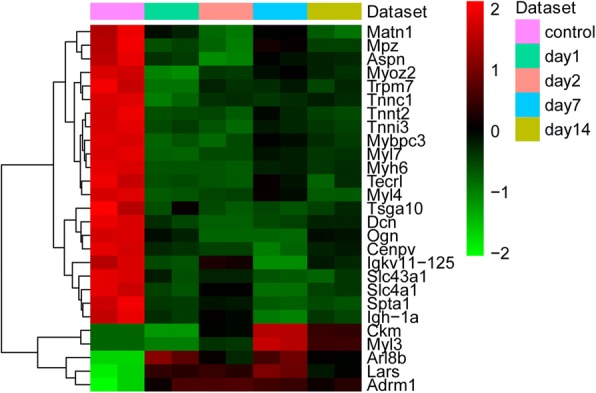
The heat map of 27 common differentially expressed proteins on day 1, 2, 7 and 14. Diagram presents the result of a two-way hierarchical clustering of 27 common differentially expressed proteins and time points. The clustering is constructed using the complete-linkage method together with the Euclidean distance. Each row represents a differentially expressed protein and each column, a time point. Differentially expressed protein clustering tree is shown on the right. The colour scale illustrates the relative level of differentially expressed protein expression: red, below the reference channel; green, higher than the reference

In this study, a proteomics analysis was used for differentially expressed protein identification in lung tissues on days 1, 2, 7 and 14 after smoke inhalation. A total of 3377 differentially expressed proteins were identified. Among these, 27 common differentially expressed proteins were obtained on days 1, 2, 7 and 14 after smoke inhalation. A Venn diagram of the differentially expressed proteins on day 1 vs day 0, day 2 vs day 0, day 7 vs day 0 and day 14 vs day 0 is shown in Fig. 3. In addition, 27 common differentially expressed proteins are listed in Table 1. Figure 4 shows the heat map of the 27 common differentially expressed proteins.

**Table 1:** Twenty seven common differentially expressed proteins

	Day 1 vs con			Day 2 vs con			Day 7 vs con			Day 14 vs con		
Gene name	Ratio	*p* value	Regulated	Ratio	*p* value	Regulated	Ratio	*p* value	Regulated	Ratio	*p* value	Regulated
Adrm1	1.65	1.89E-02	Up	1.73	5.73E-03	Up	1.69	6.66E-03	Up	1.61	1.0E-02	Up
Arl8b	1.90	4.41E-03	Up	1.55	1.51E-02	Up	1.83	3.74E-03	Up	1.59	1.2E-04	Up
Aspn	0.53	8.46E-03	Down	0.39	5.02E-03	Down	0.61	1.28E-02	Down	0.56	9.3E-03	Down
Cenpv	0.58	1.80E-03	Down	0.55	1.10E-03	Down	0.47	2.31E-03	Down	0.60	2.1E-03	Down
Ckm	0.46	1.87E-03	Down	2.00	1.85E-04	Up	3.95	2.97E-05	Up	2.59	1.2E-04	Up
Dcn	0.64	2.49E-03	Down	0.59	5.31E-04	Down	0.62	8.97E-04	Down	0.67	8.2E-04	Down
Igh-1a	0.55	7.01E-03	Down	0.63	9.74E-03	Down	0.42	3.85E-03	Down	0.53	6.1E-03	Down
Igkv11–125	0.47	4.75E-03	Down	0.66	1.16E-02	Down	0.35	2.93E-03	Down	0.56	7.1E-03	Down
Lars	1.73	4.24E-03	Up	1.73	5.17E-03	Up	1.92	3.18E-03	Up	1.57	8.1E-03	Up
Matn1	0.39	1.88E-02	Down	0.16	9.66E-03	Down	0.45	2.11E-02	Down	0.19	1.1E-02	Down
Mpz	0.47	1.22E-02	Down	0.39	9.89E-03	Down	0.63	2.29E-02	Down	0.48	1.2E-02	Down
Mybpc3	0.52	6.43E-04	Down	0.53	2.22E-03	Down	0.65	4.52E-04	Down	0.58	6.9E-04	Down
Myh6	0.37	4.17E-04	Down	0.37	4.01E-04	Down	0.48	6.89E-04	Down	0.44	7.8E-04	Down
Myl3	0.66	4.53E-04	Down	1.66	1.22E-03	Up	5.05	5.73E-04	Up	3.21	1.1E-05	Up
Myl4	0.13	4.57E-04	Down	0.19	5.86E-04	Down	0.37	2.70E-03	Down	0.13	3.7E-04	Down
Myl7	0.34	1.86E-04	Down	0.39	2.39E-04	Down	0.50	6.95E-04	Down	0.43	3.5E-04	Down
Myoz2	0.40	3.63E-04	Down	0.54	5.03E-04	Down	0.63	1.44E-03	Down	0.56	8.0E-04	Down
Ogn	0.63	1.37E-03	Down	0.52	2.67E-05	Down	0.51	3.31E-05	Down	0.64	9.5E-05	Down
Slc43a1	0.59	8.39E-03	Down	0.62	1.51E-04	Down	0.54	1.14E-04	Down	0.56	5.4E-03	Down
Slc4a1	0.54	3.27E-03	Down	0.66	3.50E-03	Down	0.48	1.55E-03	Down	0.56	2.5E-03	Down
Spta1	0.61	6.15E-03	Down	0.66	7.99E-03	Down	0.56	4.69E-03	Down	0.58	5.3E-03	Down
Tecrl	0.50	5.89E-03	Down	0.48	5.36E-03	Down	0.62	1.27E-02	Down	0.50	1.6E-02	Down
Tnnc1	0.29	1.50E-03	Down	0.44	1.63E-04	Down	0.43	2.31E-04	Down	0.46	1.2E-03	Down
Tnni3	0.39	1.00E-03	Down	0.32	1.23E-03	Down	0.46	1.18E-03	Down	0.40	7.2E-04	Down
Tnnt2	0.41	1.96E-03	Down	0.41	3.17E-03	Down	0.52	7.71E-03	Down	0.43	2.2E-03	Down
Trpm7	0.40	4.68E-03	Down	0.53	8.78E-03	Down	0.54	9.93E-03	Down	0.51	9.9E-03	Down
Tsga10	0.54	4.55E-02	Down	0.46	1.52E-02	Down	0.47	1.59E-02	Down	0.51	2.0E-02	Down

*p*-value ≤0.05 was considered significant

### WGCNA co-expression network analysis

**Fig. 5:**
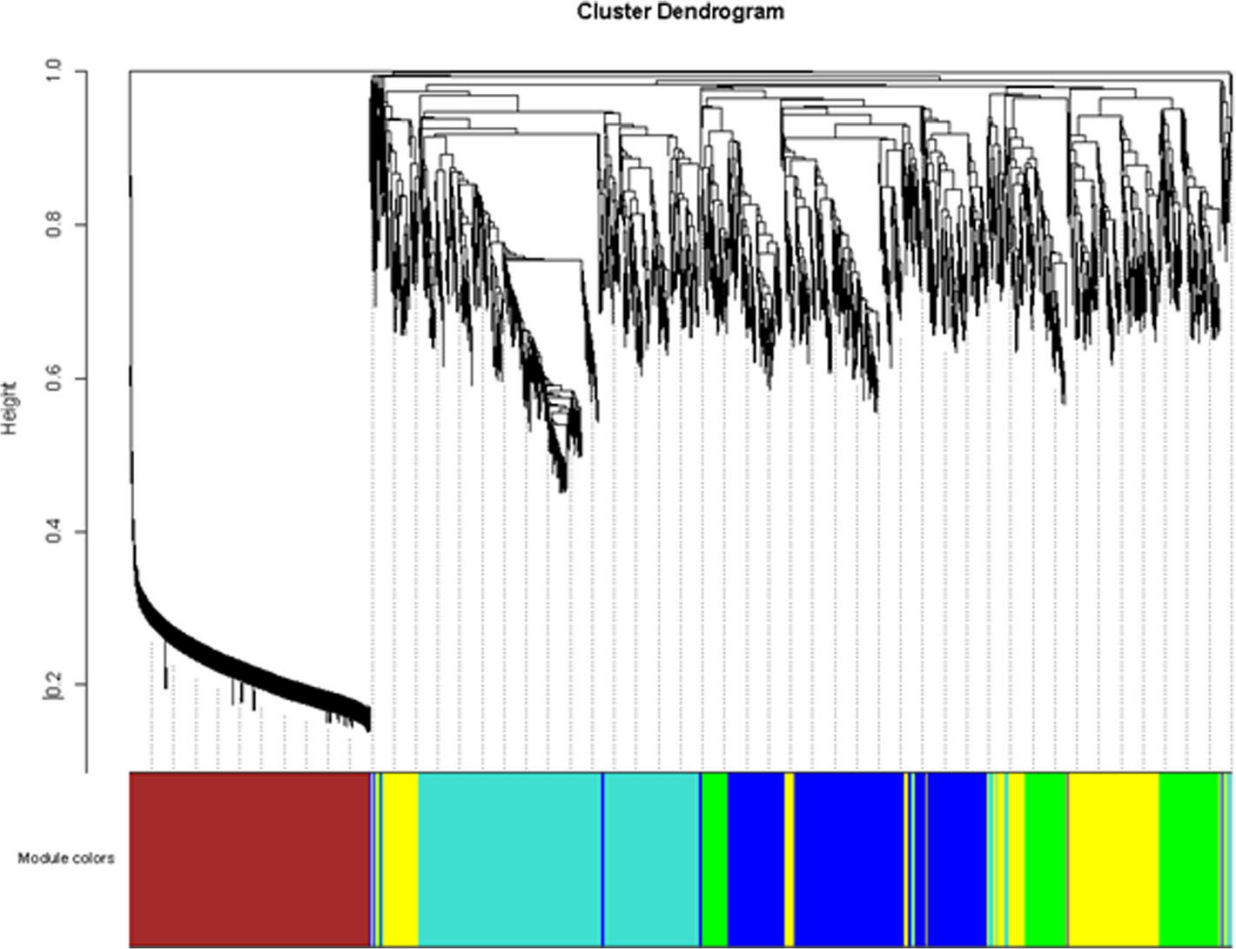
Clustering results of modules in proteome data. Genes in modules are marked with different colors (green, blue, yellow, brown, turquoise and grey). The lower panel shows colors designated for each module

**Fig. 6:**
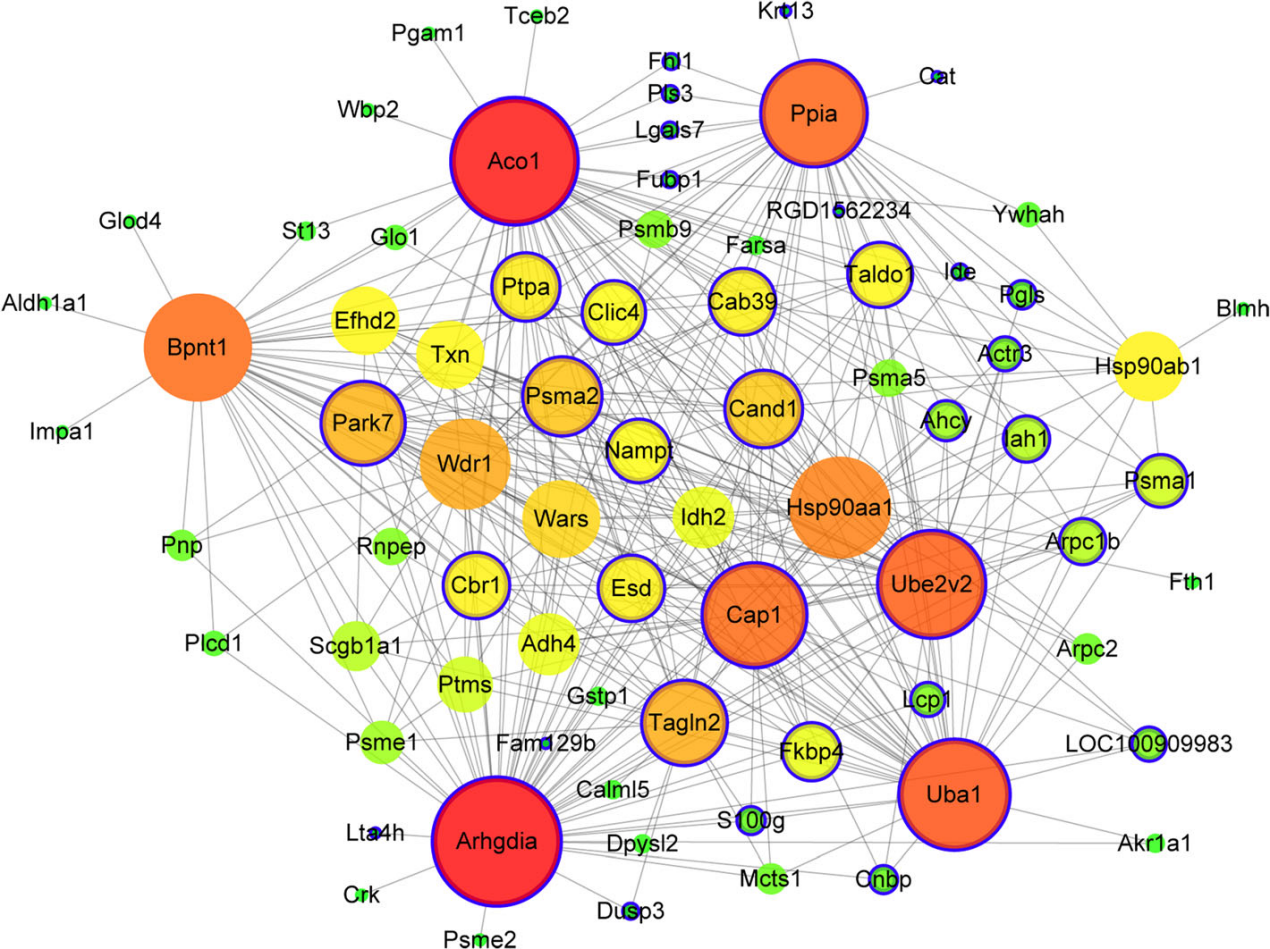
The visualization of the turquoise module in the WGCNA co-expression network. The larger circle represents the higher degree protein. The blue border indicates hub protein

In the WGCNA co-expression network analysis, a total of 6 modules (green, blue, yellow, brown, turquoise and grey) were identified (Fig. 5). To further explore the correlation between the modules and the smoke inhalation time, a correlation analysis was performed, and the modules with a correlation coefficient > 0.8 and *p* < 0.001 were considered to be related. The correlation analysis showed that the turquoise module (which had 909 proteins) was associated with smoke inhalation time, and the other modules were not related to smoke inhalation time (*p* > 0.05), as shown in Table 2. In addition, the visualization of the turquoise module, which consists of 76 nodes and 370 edges, is shown in Fig. 6.

**Table 2:** The correlation analysis between modules and smog inhalation time

Module	Correlation coefficient	*p* value
green	−0.35	0.32
blue	−0.09	0.81
yellow	−0.33	0.35
brown	0.0029	0.99
turquoise	0.88	0.00086
grey	−0.031	0.93

### Functional annotation of the turquoise module

**Fig. 7:**
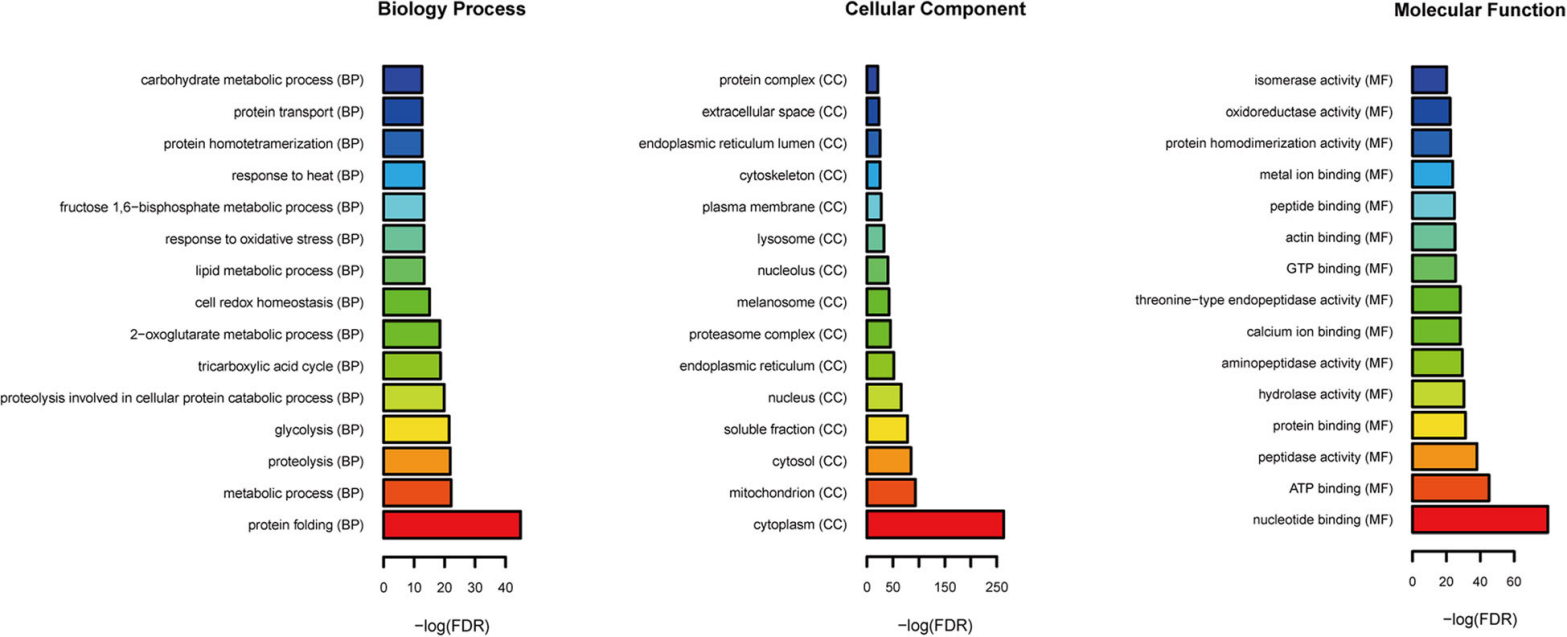
GO enrichment analysis of top 15 turquoise module

According to the GO enrichment analysis (Fig. 7), protein folding, metabolic processes and proteolysis were the most significantly enriched biological processes; the cytoplasm, mitochondria, and cytosol were the most significantly enriched cellular components; nucleotide binding, ATP binding and peptidase activity were the most significantly enriched molecular functions. In addition, based on the KEGG enrichment analysis, proteasome, protein processing in endoplasmic reticulum, lysosome and glycolysis/gluconeogenesis were enriched signaling pathways (Table 3).

**Table 3:** Top 15 most significantly enriched KEGG pathways of DEGs in the turquoise module

KEGGID	Term	Count	FDR	Symbols
03050	Proteasome	27	2.21E-31	Psmb9,Psma6,Psmb1,Psma3,Psme2,Psmc2,Psmb2,Psmc6,Psmd11,Psmd6,Psma1,Psmc3,Psma4,Psme1,Psmc1,Psmc5,Psmd2,Psmb3,Psmb4,Psmb10,Psmd13,Psmd1,Psma5,Psma7,Psmd4,Psmc4,Psmd7
04141	Protein processing in endoplasmic reticulum	28	3.00E-15	Bcap31,Dnajb2,Sar1a,Sel1l,Hsp90aa1,Prkcsh,Pdia3,Rad23b,Dnajb1,Hsp90b1,Erp29,Sec61a1,Skp1,Pdia4,Stt3a,Calr,Man1a1,Nsfl1c,Nploc4,Ganab,Dnajc10,P4hb,Ckap4,Lman2,Hsp90ab1,Hsph1,Uggt1,Vcp
04142	Lysosome	24	6.97E-15	Man2b1,Naga,Ctsc,Gm2a,Lipa,Lgmn,Ctsd,Abcb9,Fuca1,Glb1,Lamp2,Ctsa,Igf2r,Ap3d1,Atp6v1h,Asah1,Psap,Gns,Ctsb,Tpp1,Ppt1,Napsa,Npc2,Ctsh
00010	Glycolysis/Gluconeogenesis	18	2.84E-14	Ldha,Pgm1,Dld,Aldh9a1,Eno3,Aldoc,Akr1a1,Aldh7a1,Tpi1,Aldoa,Aldh1a1,Pck2,Gapdh,Dlat,Pfkp,Pfkm,Pgk1,Pfkl
00020	Citrate cycle (TCA cycle)	14	8.01E-14	Idh1,Idh3g,Acly,Dld,Dlst,Suclg1,Aco2,Ogdh,Pck2,Idh2,Sdhb,Mdh2,Aco1,Dlat
04145	Phagosome	23	3.91E-11	Rac1,RT1-Bb,Tubb5,RT1-Da,Atp6v1a,Sec61a1,Atp6v1c1,Lamp2,Calr,RT1-DMa,Atp6v1e1,Mpo,Tap1,Atp6v1h,Itga2,RT1-Db1,Tubb2a,Atp6v1b2,Itga5,Cd14,Eea1,Dync1li1,Sftpa1
00970	Aminoacyl-tRNA biosynthesis	13	8.60E-11	Vars,Dars,Yars,Wars,Sars,Farsa,Gars,Nars,Tars,Farsb,Hars,Kars,Aars
00620	Pyruvate metabolism	11	4.75E-09	Acaca,Ldha,Dld,Aldh9a1,Aldh7a1,Aldh1a1,Pck2,Akr1b1,Mdh2,Glo1,Dlat
00480	Glutathione metabolism	12	1.33E-08	Idh1,Lap3,Txndc12,Gsr,Gss,Mgst1,Gpx7,Gsta4,Gstm1,Idh2,Gstm2,Gstp1
04612	Antigen processing and presentation	14	3.14E-08	Hsp90aa1,RT1-Bb,Psme2,RT1-Da,Pdia3,Lgmn,Calr,RT1-DMa,Tap1,Hspa4,RT1-Db1,Psme1,Ctsb,Hsp90ab1
05100	Bacterial invasion of epithelial cells	13	8.79E-08	Rac1,Arpc1b,Vcl,Arpc5l,Arpc2,Fn1,Elmo1,Dnm2,Arpc3,Itga5,Cav2,Arpc5,Crk
00280	Valine, leucine and isoleucine degradation	11	1.10E-07	Dld,Pcca,Aldh9a1,Aldh7a1,Acads,Hmgcl,Bcat2,Aldh1a1,Acadsb,Hadh,Acaa2
00030	Pentose phosphate pathway	9	1.26E-07	Pgm1,Aldoc,Tkt,Taldo1,Aldoa,Pfkp,Pgls,Pfkm,Pfkl
04666	Fc gamma R-mediated phagocytosis	13	8.76E-07	Rac1,Arpc1b,Marcks,Arpc5l,Arf6,Dstn,Arpc2,Cfl1,Dnm2,Prkcd,Arpc3,Arpc5,Crk
00052	Galactose metabolism	7	7.01E-06	Pgm1,Glb1,Ugp2,Akr1b1,Pfkp,Pfkm,Pfkl

### Hub protein identification in the turquoise module

**Fig. 8:**
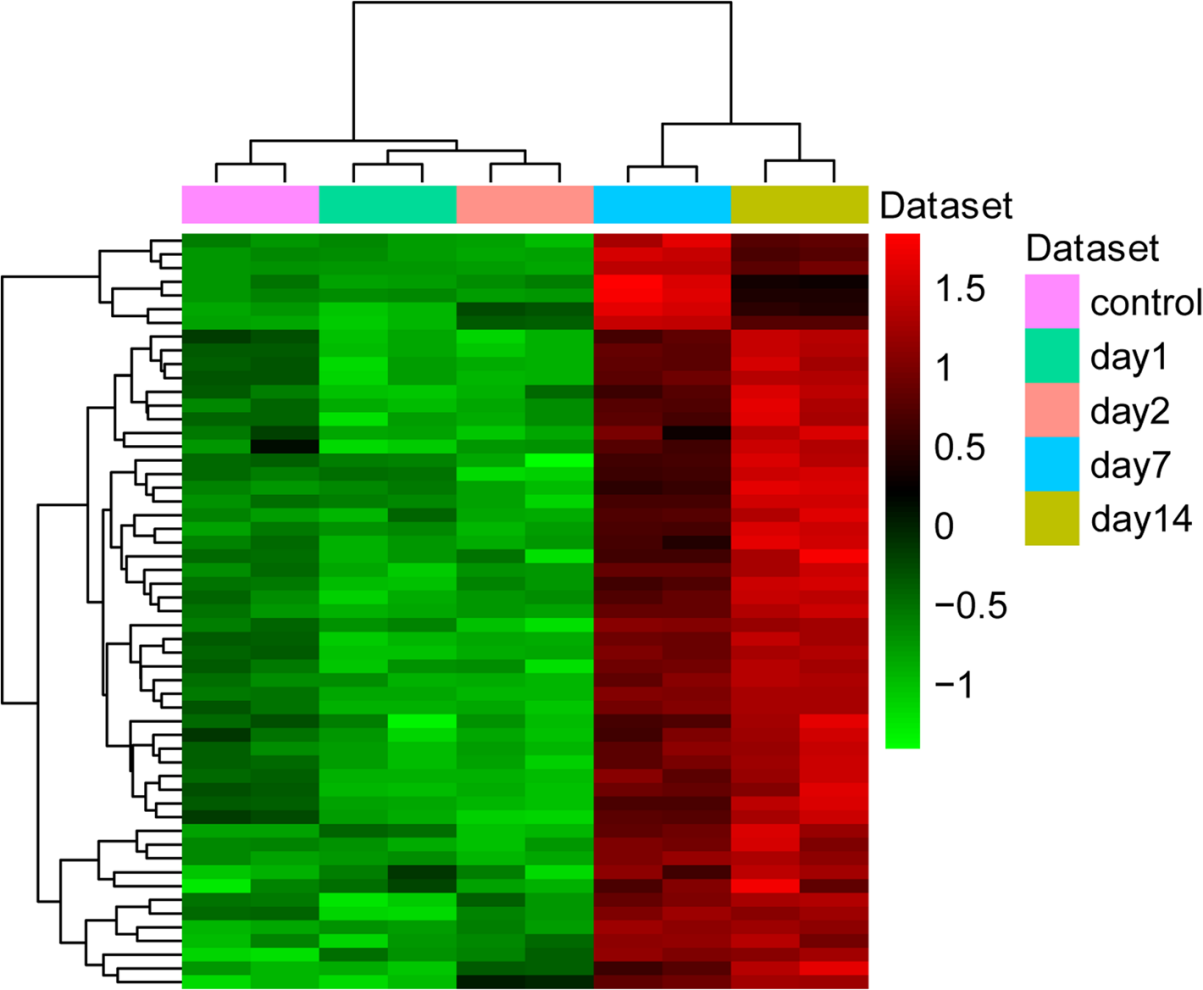
The heat map of 55 hub proteins in the turquoise module. Diagram presents the result of a two-way hierarchical clustering of 55 hub differentially expressed proteins and time points. The clustering is constructed using the complete-linkage method together with the Euclidean distance. Each row represents a hub differentially expressed protein and each column, a time point. The hub differentially expressed protein clustering tree is shown on the right. The colour scale illustrates the relative level of hub protein expression: red, below the reference channel; green, higher than the reference

To further identify important proteins, we screened the hub proteins in the turquoise module. A total of 55 hub proteins were identified (Table 4). Additionally, the heat map of 55 hub proteins is shown in Fig. 8. In Fig. 8, 55 differentially expressed hub proteins were significantly upregulated on day 7 and day 14 (middle-late stage) after smoke inhalation, which suggests that these proteins may be associated with lung injury repair from smoke inhalation.

### Injury repair-related differentially expressed proteins analysis in the early and middle-late stages of zinc chloride smoke inhalation

**Fig. 9:**
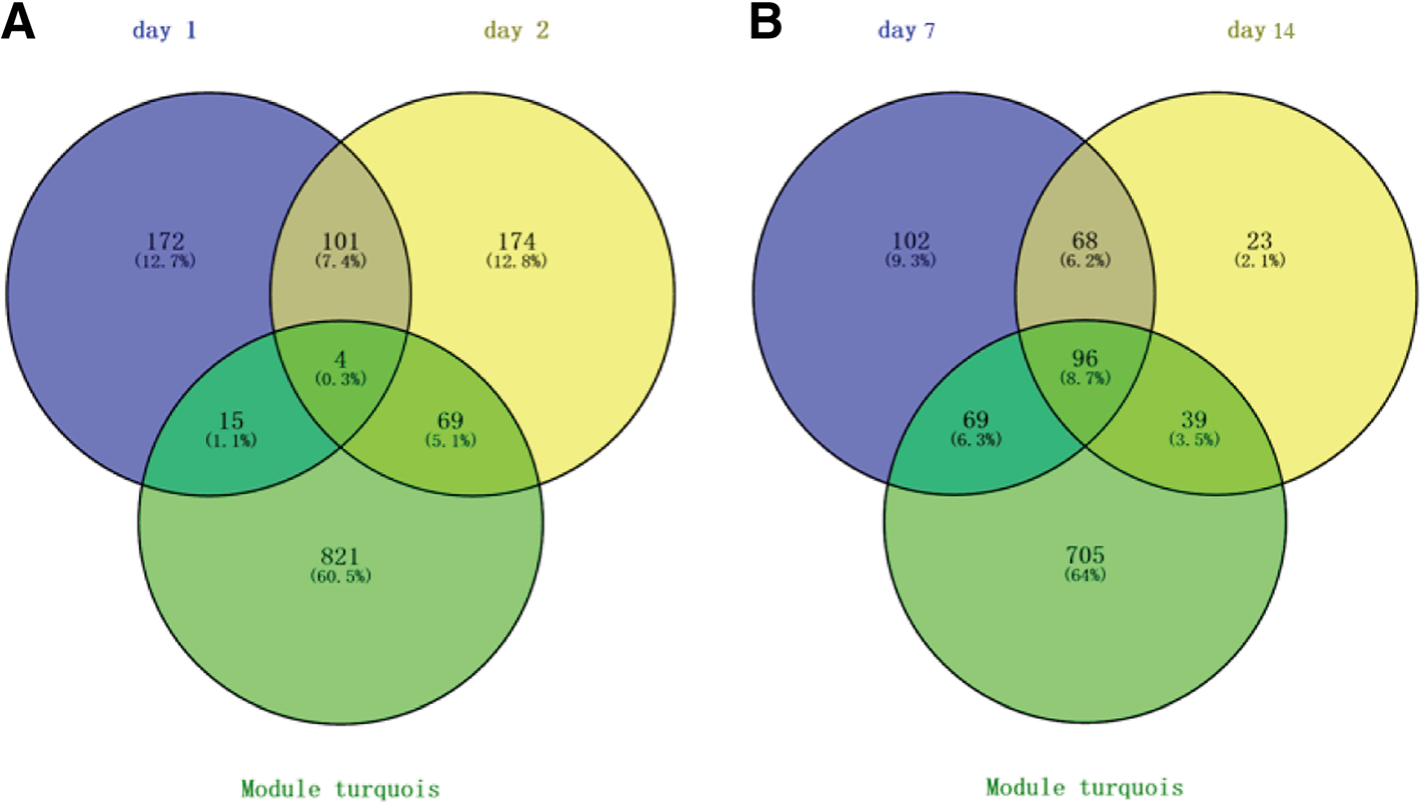
The Venn diagram of related differentially expressed proteins analysis in the early and middle-late stage of smoke inhalation. **a** early stage of smoke inhalation. **b** middle-late stage of smoke inhalation

The histopathology results showed obvious lung injury on day 1 and day 2 after zinc chloride smoke inhalation. Therefore, it is necessary to find smoke inhalation lung injury repair-related differentially expressed proteins and related signalling pathways in the early and middle-late stages of smoke inhalation. A total of 4 differentially expressed proteins, including Myl3, Ckm, Adrm1 and Igfbp7, were identified after overlapping the early stage of injury with the turquoise module after smoke inhalation. In addition, a total of 96 proteins were identified after overlapping the middle-late stage of injury from smoke inhalation with the turquoise module. A Venn diagram of related differentially expressed proteins in the early and middle-late stages of smoke inhalation is shown in Fig. 9. In addition, after KEGG enrichment analysis of these 96 differentially expressed proteins, we found that the lysosome, glycolysis/gluconeogenesis pathway (involving Eno3 and Tpi1), pentose phosphate pathway, amino sugar and nucleotide sugar metabolism, dust collection duct acid secretion, fructose and mannose metabolism, starch and sucrose metabolism and citrate cycle (TCA cycle) were the enriched signalling pathways (Table 5).

### PPI network

**Fig. 10:**
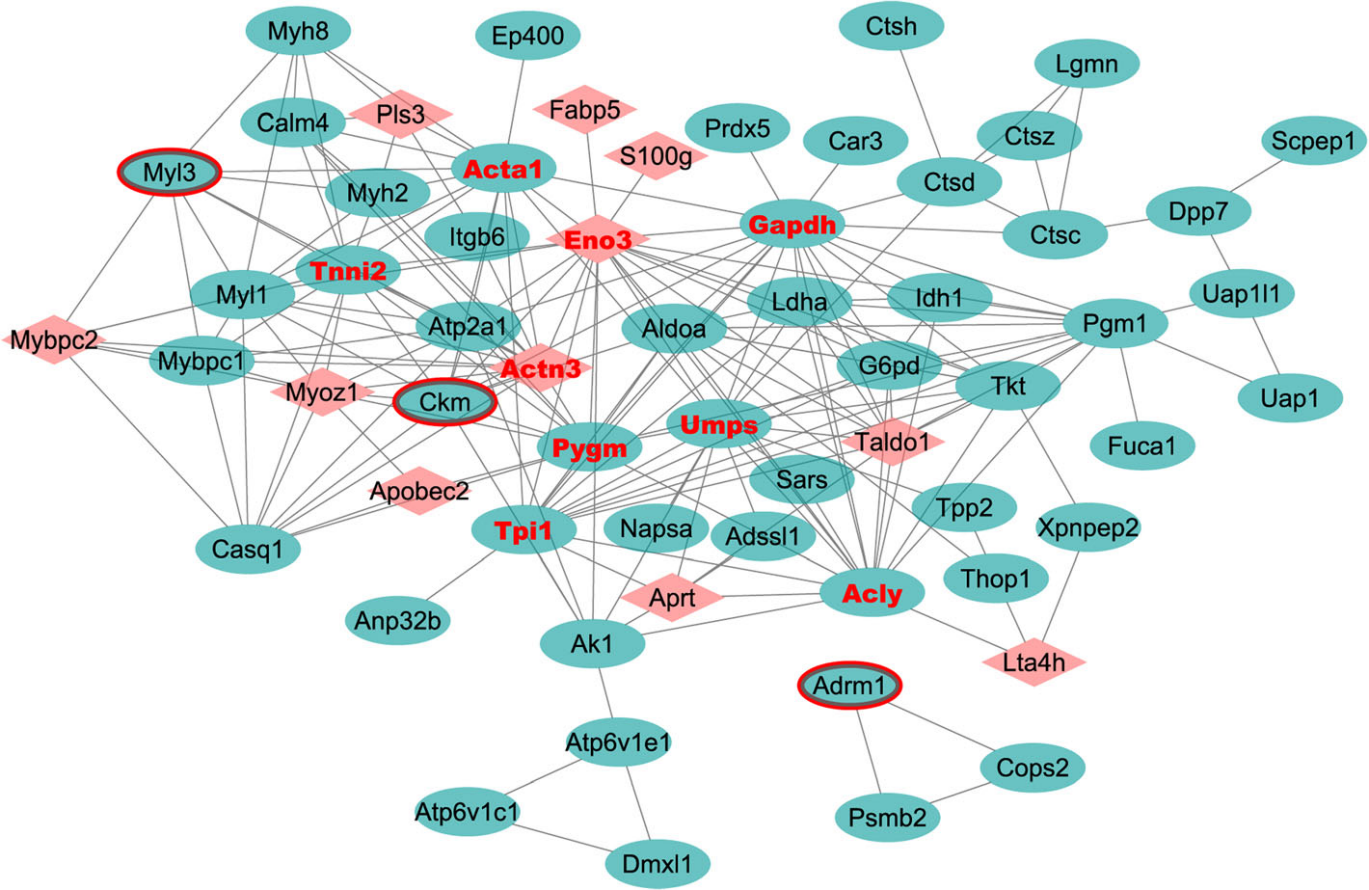
PPI networks of key differentially expressed proteins in the early and middle-late stage of smoke inhalation. Red triangle represents the hub protein in the WGCNA analysis; Red border represents the common differentially expressed proteins on day 1, 2, 7 and 14; Red font represents the protein with a high degree in the PPI network

The PPI network of identified key differentially expressed proteins in the early stage (4 differentially expressed proteins were identified after overlapping the early stage of smoke inhalation with the turquoise module) and middle-late stage (96 differentially expressed proteins were identified after overlapping the middle-late of smoke inhalation with the turquoise module) of smoke inhalation was determined by Cytoscape software. There were 61 nodes and 205 edges in the network (Fig. 10). Interestingly, Eno3 (degree = 20), Gapdh (degree = 19), Acly (degree = 17), Umps (degree = 17), Tpi1 (degree = 15), Pygm (degree = 15), Tnni2 (degree = 15), Actn3 (degree = 15), and Acta1 (degree = 15) were proteins with a high degree of differential expression.

### Immunohistochemistry validation of PARK7

**Fig. 11:**
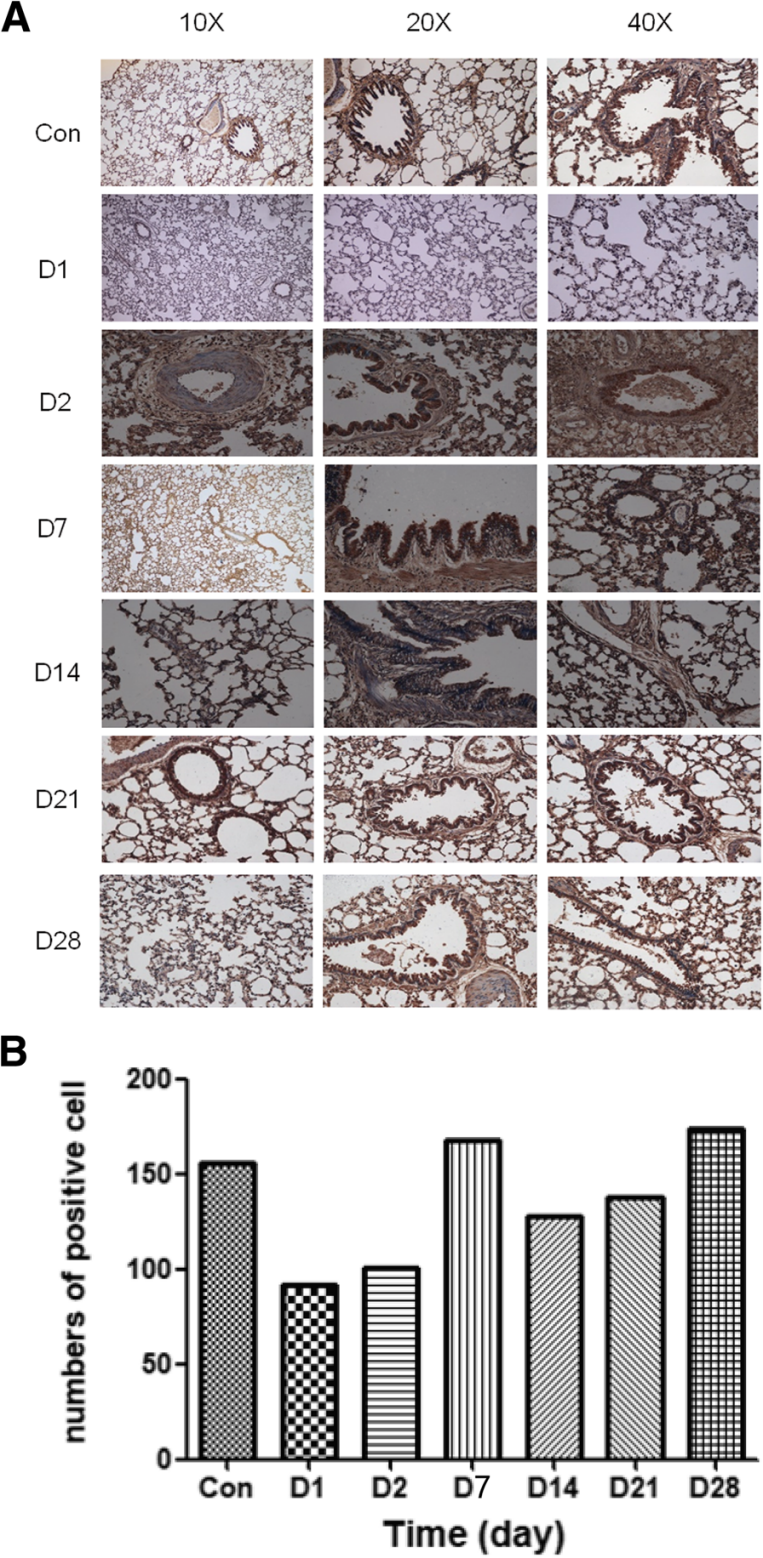
IHC staining of PARK7. **a** PARK7 protein expression level detected by immunohistochemistry and photographs was amplified 10×, 20× and 40× multiples. **b** The statistics of numbers of positive staining cell

In this study, PARK7, a differentially expressed protein after day 14 of smoke inhalation, was selected for IHC analysis on days 1, 2, 8, 14, 21 and 28 (Fig. 11). The brown colour represents the positive staining of PARK7. The expression of PARK7 was mainly located in the cytoplasm and the nucleus. Moreover, the density of PARK7 staining in the nucleus increased over time after smoke inhalation. The strong positive staining signal (especially in the control group) was located in the bronchial epithelial cells and the alveolar epithelial cells. The validation result of PARK7 was consistent with the results of the proteomics analysis.

### Western blot analysis of PARK7 and FABP5

**Fig. 12:**
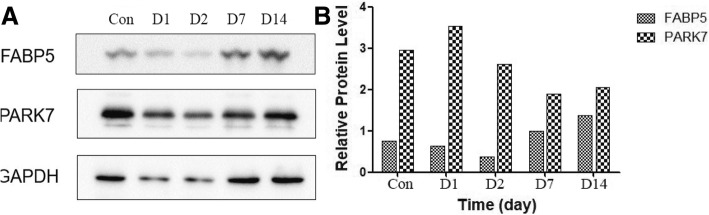
Densitometric analysis of PARK7 and FABP5 protein expression after normalization to GAPDH. **a** PARK7 and FABP5 protein expression level detected by western blot. **b** The statistics analysis of protein band. Student t-test was used and all data were reported as mean ± SD

In this study, the differentially expressed proteins PARK7 and FABP5 on day 14 after smoke inhalation were selected for WB analysis on days 1, 2, 7 and 14 (Fig. 12). The expression of PARK7 was highest in the control group and decreased with the smoke inhalation time. The expression of FABP5 was highest on day 14. The validation result is consistent with the proteomics analysis.

**Table 4:** Fifty five hub proteins in the turquoise module

Protein	Coefficient.Time	p.Time	Coefficient.turquoise	p.turquoise
Aars	0.90	3.37E-04	0.90	3.63E-04
Aco1	0.90	3.25E-04	0.98	7.60E-07
Actn3	0.97	2.02E-06	0.92	1.39E-04
Actr3	0.95	2.61E-05	0.96	7.56E-06
Adk	0.98	9.53E-07	0.90	3.76E-04
Ahcy	0.96	1.06E-05	0.94	4.02E-05
Apobec2	0.97	2.29E-06	0.91	2.60E-04
Aprt	0.90	3.38E-04	0.97	4.13E-06
Arhgdia	0.92	1.50E-04	0.95	1.96E-05
Arpc1b	0.96	1.58E-05	0.95	1.94E-05
Cab39	0.90	3.69E-04	0.96	1.64E-05
Cand1	0.92	2.03E-04	0.97	4.51E-06
Cap1	0.91	2.12E-04	0.96	1.56E-05
Cat	0.96	6.79E-06	0.94	5.44E-05
Cbr1	0.90	3.38E-04	0.93	1.01E-04
Clic4	0.92	1.44E-04	0.95	3.05E-05
Cnbp	0.91	2.98E-04	0.91	2.91E-04
Cndp2	0.94	5.04E-05	0.95	3.64E-05
Dusp3	0.92	1.56E-04	0.91	2.73E-04
Eno3	0.94	6.44E-05	0.91	2.59E-04
Esd	0.92	1.47E-04	0.92	1.86E-04
Fabp5	0.96	1.67E-05	0.97	3.15E-06
Fam129b	0.96	1.10E-05	0.92	1.34E-04
Fhl1	0.93	7.42E-05	0.98	2.58E-07
Fkbp4	0.94	4.15E-05	0.91	2.41E-04
Fubp1	0.92	1.32E-04	0.97	2.19E-06
Gars	0.91	2.89E-04	0.97	3.48E-06
Iah1	0.94	5.64E-05	0.93	7.31E-05
Ide	0.97	2.21E-06	0.94	4.85E-05
Krt13	0.95	3.25E-05	0.98	6.44E-07
Lcp1	0.97	3.50E-06	0.93	9.37E-05
Lgals7	0.94	5.41E-05	0.97	3.31E-06
LOC100909983	0.95	2.22E-05	0.92	1.54E-04
Lta4h	0.98	1.13E-06	0.92	1.58E-04
Mapk15	0.92	2.03E-04	0.99	5.66E-08
Mtpn	0.94	6.40E-05	0.90	3.20E-04
Mybpc2	0.94	3.94E-05	0.96	1.19E-05
Myoz1	0.93	1.05E-04	0.93	7.84E-05
Nampt	0.93	1.11E-04	0.91	2.44E-04
Park7	0.92	1.33E-04	0.94	4.58E-05
Pgls	0.93	7.55E-05	0.98	1.38E-06
Pls3	0.92	1.89E-04	0.96	1.68E-05
Ppia	0.91	2.08E-04	0.99	9.95E-08
Psma1	0.91	2.73E-04	0.97	4.48E-06
Psma2	0.92	1.76E-04	0.96	8.57E-06
Psmc4	0.92	1.58E-04	0.92	1.80E-04
Ptpa	0.91	3.16E-04	0.94	4.96E-05
RGD1562234	0.95	3.02E-05	0.97	2.06E-06
S100 g	0.97	2.12E-06	0.91	3.16E-04
Set	0.92	1.76E-04	0.94	5.02E-05
Tagln2	0.94	5.04E-05	0.94	6.72E-05
Taldo1	0.95	3.36E-05	0.95	2.53E-05
Tprkb	0.91	2.87E-04	0.97	2.96E-06
Uba1	0.92	1.75E-04	0.97	5.91E-06
Ube2v2	0.91	2.34E-04	0.97	2.26E-06

**Table 5:** KEGG analysis of 96 differentially expressed proteins in the middle-late stage of inhalation lung injury

KEGG ID	Term	Count	FDR	Symbols
04142	Lysosome	8	1.99E-08	Ctsc,Gm2a,Lgmn,Ctsd,Fuca1,Napsa,Npc2,Ctsh
00010	Glycolysis/Gluconeogenesis	6	1.84E-07	Ldha,Pgm1,Eno3,Tpi1,Aldoa,Gapdh
00030	Pentose phosphate pathway	4	1.18E-05	Pgm1,Tkt,Taldo1,Aldoa
00520	Amino sugar and nucleotide sugar metabolism	3	2.94E-03	Pgm1,Uap1,Uap1l1
04966	Collecting duct acid secretion	2	2.12E-02	Atp6v1c1,Atp6v1e1
00051	Fructose and mannose metabolism	2	2.16E-02	Tpi1,Aldoa
00500	Starch and sucrose metabolism	2	2.18E-02	Pgm1,Pygm
00020	Citrate cycle (TCA cycle)	2	2.37E-02	Idh1,Acly

*FDR* false discovery rate

## Discussion

Since the first case of smoke bomb inhalation injury was reported in 1945, cases of smoke-induced inhalation lung injuries have been continuously reported [20]. Zinc chloride is often used for battle, military exercises, and fire-fighting training [21]. Zinc chloride aerosols are highly hygroscopic in the respiratory tract. Both the inhalation of the smoke and zinc chloride lead to pulmonary oedema, alveolitis in the early phase, late-phase pulmonary fibrosis, and often fatal acute respiratory distress syndrome in confined spaces [20, 22]. Smoke inhalation injury has a complex pathogenesis, and there is no specific treatment. Current animal models for studying inhaled lung injuries mainly include rats, ovines and rabbits [23–25]. Rats are easy to breed and are easy to develop into model animals for studying various diseases. Zhu et al. reported that the rat model of inhaled smoke is particularly suitable for studying the effects of long-term inhalation of smoke on lung injury [8]. With high-throughput analysis, the emergence of profiles has become an effective method to identify differentially expressed genes and proteins in a variety of diseases. These profiles help to explore pathogenesis and to develop biomarkers. A high-throughput sequencing analysis has suggested that circRNAs were differentially expressed during acute lung injury induced by smoke inhalation in rat lung tissue [23]. To our knowledge, this is the first time that differential proteins have been identified after an inhaled lung injury in rats using proteomic analysis. Here, we established a model of rat zinc chloride smoke inhalation to define proteins associated with inhaled lung injury. In this study, we explored the histopathology of rat lungs after zinc chloride smoke inhalation. On day 1 of smoke inhalation, lung injury occurred. On day 7 after smoke inhalation, injury repair began. On day 14 after smoke inhalation, repair to the injury was obvious. This suggested that smoke inhalation had an effect on the lungs. In addition, after the WGCNA co-expression network analysis, we finally obtained a total of 55 differentially expressed hub proteins. Interestingly, 55 hub proteins were upregulated on days 7 and 14 after smoke inhalation, which suggests that these proteins may be associated with injury repair. Based on this, we further analysed the differentially expressed proteins related to lung injury repair in the early (day 1 and day 2) and middle-late (day 7 and day 14) stages after smoke inhalation. Finally, a total of 4 proteins, including Ckm, Adrm1, Igfbp7 and Myl3, were identified in the early stages of lung injury after smoke inhalation.

Creatine kinase muscle (Ckm) is an early, sensitive tissue leakage biomarker of skeletal muscle injury [26]. Adhesion regulating molecule 1 (Adrm1) encodes the integral plasma membrane protein involved in proteolysis and deubiquitination [27–30]. It has been reported that Adrm1 is downregulated in ageing-associated idiopathic pulmonary fibrosis [31]. Furthermore, downregulation of Adrm1 at the mRNA and protein levels leads to slower wound-healing [32]. Insulin-like growth factor binding protein 7 (Igfbp7), a cell cycle arrest biomarker, is released in the earliest stages of acute kidney injury, which has been suggested to be an important predictor of acute kidney injury following cardiac surgery [33–35]. We hypothesize that Ckm, Adrm1 and Igfbp7 may also be involved in the early repair of lung injury by zinc chloride smoke inhalation.

Myosin light polypeptide 3 (Myl3) is a biomarker for monitoring muscle injury and recovery [26]. It has been found that Myl3 is significantly downregulated in spinal cord injuries [36]. In addition, it is a causal gene for hypertrophic cardiomyopathy [37]. It has been demonstrated that Myl3 is a plasma marker of cardiac injury [38]. In this study, we found that Myl3 was differentially expressed in the early stages of lung injury after smoke inhalation. It has been found that cardiac dysfunction results from the inhalational of Cl_2_; this exposure may result in pulmonary hypertension due to severe lung injury [39]. In addition, cardiac output varies in patients with acute lung injury with or without right ventricular dilatation [40]. We speculate that Myl3 may be involved in lung injury repair in the early stage after smoke inhalation.

Based on the KEGG enrichment analysis, the proteasome, protein processing in endoplasmic reticulum, the lysosome and glycolysis/gluconeogenesis were enriched signalling pathways and organelles. Aldehyde dehydrogenase 7A1 (ALDH7A1), Enolase 3 (Eno3) and triosephosphate isomerase 1 (Tpi1) were enriched in the glycolysis/gluconeogenesis signalling pathways. ALDH7A1 is a member of the ALDH superfamily. The primary function of these enzymes is the detoxification of endobiotic and xenobiotic aldehyde compounds into their corresponding weak carboxylic acids. Giacalone et al. reported that ALDH7A1 is the potential target of ALDH enzymes in the treatment of lung cancer [41]. Eno3 is a highly expressed protein in the lungs [42]. In guinea pig lungs, Eno3 is involved in glycolysis/gluconeogenesis [43]. It has been reported that Eno3 is upregulated in traumatic brain injury [44]. The expression of triosephosphate isomerase 1 (Tpi1) is low in smoking-induced emphysema lung models [45]. It is worth mentioning that Tpi1 is involved in the glycolysis or gluconeogenesis pathway in acute lung injury [46]. The lungs depend on circulation for glucose acquisition. Therefore, the glycolysis/gluconeogenesis signalling pathway is important in the physiological activities of the lungs. Our results suggest that ALDH7A1, Eno3 and Tpi1 may be involved in glucose metabolism in the middle-late stage of lung injury repair.

According to the PPI, we found highly expressed proteins, including Gapdh, Acly, Tnni2, Acta1, Actn3, Pygm, Eno3 and Tpi1. It is noted that all these proteins were differentially expressed in the middle-late stage of injury after smoke inhalation. It has been shown that glyceraldehyde-3-phosphate dehydrogenase (Gapdh) has an anti-inflammatory function in the lipopolysaccharide-induced severe acute lung injury mouse model [47]. ATP citrate lyase (Acly), a lipid biosynthesis transcript, plays an important role in chronic obstructive pulmonary disease [48]. It has been pointed out that Acly is downregulated in acrolein-induced acute lung injury [49]. Troponin I, skeletal fast 2 (Tnni2) plays a crucial role in lung veins [50]. In rats, Tnni2 is involved in angiogenesis in the early stage of skin incision wounds [51]. Actin, alpha 1 skeletal muscle (Acta1) is a representative marker of myocardial adaptive response to damage [52]. Significantly, Acta1 is a differentially regulated gene in nickel-induced acute lung injury [53]. Muscle glycogen phosphorylase (Pygm) is involved in glycogen metabolism [54]. The homozygous mutation in the Pygm gene leads to glycogen metabolism disorder in patients with acute kidney injury [55].

Parkinson’s disease 7 (Park7) is autosomal recessive with an early onset and is a metabolic enzyme. The expression of Park7 is downregulated in lung development [56]. It has been found that Park7 is downregulated in asbestos-exposed epithelial and mesothelial lung cell lines [57]. In this study, we found that Park7 was upregulated on day 14 after zinc chloride smoke inhalation. In addition, the immunohistochemistry results showed that the expression of PARK7 was highest in the control group and decreased with time after zinc chloride smoke inhalation.

In addition to the proteins listed above, we also found some differentially expressed proteins (such as Lta4h and Fabp5) in the middle-late stages of injury after zinc chloride smoke inhalation. It is noted that Lta4h is a hub protein in the turquoise module of WGCNA analysis (Table 4). Leukotriene A4 hydrolase (Lta4h) polymorphisms are associated with baseline lung function [58]. It has been found that Lta4h is related to chronic obstructive pulmonary disease [59]. The epidermal fatty acid binding protein 5 (Fabp5) is expressed in lung tissues, especially in alveolar macrophages [60]. A previous study indicated that Fabp5 was downregulated in the airway epithelial cells of smokers with chronic obstructive pulmonary disease [61]. In addition, a microarray experiment showed the expression of Fabp5 in a mouse lung during nickel-induced lung injury [62]. Our WB results showed that the expression of FABP5 was the highest on day 14, which was consistent with the proteomics analysis. In addition, 10 differentially expressed proteins, including Gapdh, Acly, Tnni2, Acta1, Actn3, Pygm, Eno3, Tpi1, Lta4h, Fabp5 and the glycolysis/gluconeogenesis signalling pathway, may play roles in the middle-late stage of zinc chloride smoke inhalation lung injury repair. However, there are limitations to our study. The biological functions of the identified differentially expressed proteins were not thoroughly investigated. Some in vivo or in vitro experiments, such as cell experiments, are needed.

## Conclusion

We first analysed differentially expressed proteins on days 1, 2, 7 and 14 after zinc chloride smoke inhalation in rats. Then, we further identified lung injury repair-related differentially expressed proteins in the early and middle-late stages of lung injury. The identified key proteins and related signalling pathways may play crucial roles in smoke inhalation lung injury repair.

## Abbreviations

FDR: False discovery rate
GO: Gene Ontology
H&E: Ematoxylin and eosin
IHC: Immunohistochemistry
Itraq: Isobaric tags for relative and absolute quantization
KEGG: Kyoto Encyclopedia of Genes and Genomes
PPI: Protein-protein interaction
WB: Western blot
WGCNA: Weighted gene co-expression network analysis
